# The complete chloroplast genome sequence of *Ilex chinensis* Sims. (Aquifoliaceae), a folk herbal medicine plant in China

**DOI:** 10.1080/23802359.2021.1904800

**Published:** 2021-03-26

**Authors:** Yanwei Zhou, Naiwei Li, Hong Chen, Xinran Chong, Yunlong Li, Xiaoqing Lu, Ting Zhou, Fan Zhang

**Affiliations:** Jiangsu Key Laboratory for the Research and Utilization of Plant Resources, Institute of Botany, Jiangsu Province and Chinese Academy of Sciences, Nanjing, PR China

**Keywords:** *Ilex chinensis*, complete chloroplast genome, phylogenetic analysis

## Abstract

The complete chloroplast (cp) genome of *Ilex chinensis*, an important economic plant with ornamental and ecological values, was sequenced to investigate its phylogenetic relationship. The entire cp genome of *I. chinensis* was 157,885 bp in length with 37.61% overall GC content, including a large single-copy (LSC) region of 87,289 bp, and a small single-copy (SSC) region of 18,388 bp, which were separated by a pair of inverted repeats (IRs) of 52,208 bp. The cp genome contained 135 genes, including 90 protein-coding genes, 37 *tRNA* genes, and eight *rRNA* genes. Phylogenetic analysis based on whole cp genome sequences showed that *I. chinensis* was closely related to *I. szechwanensis* and *I. viridis* species.

*Ilex* L. is the only living genus in the monogeneric family Aquifoliaceae that contains 600 species varying in leaf morphologies (Yao et al. 2016). *Ilex chinensis* Sims. (synonym, *I. purpurea* Hassk.) is a commonly used traditional Chinese medicine herb, which is widely spread in south part of China (Li, Xia, et al. 2018). The dried leaves of this plant are known in China as ‘Si ji qing’ used to treat acute laryngopharyngitis, bronchitis, dysentery, burns, and as an external treatment for skin ulcer in China (College 1977). At present, most of the studies of *I. chinensis* focused on the chemical composition and pharmacological effects. The chloroplast is essential organelles for photosynthesis and carbon fixation in green plants (Neuhaus and Emes 2000). Due to its relatively conserved traits (Li, Zhang, et al. 2018), chloroplast genome sequences are becoming a very useful tool in researching DNA barcode to distinguish closely related species, plant evolution, and phylogeny studies (Wambugu et al. 2015). Moreover, obtaining more information of species’ complete chloroplast genome, will provide more effective information for genetic identification and conservation of valuable features, then improving phylogenetic analysis of closely related species (Daniell et al. 2016; Wang 2017). In this article, we characterized the complete chloroplast genome sequence of *I. chinensis* with bioinformatics analysis, which would be provided basic genetic resource for *Ilex* hybrids with other species in the *Ilex* genus.

Fresh leaves of *I. chinensis* were collected from Nanjing Botanical Garden, Mem. Sun Yat-sen (118°49′55″E, 32°3′32″N), Nanjing, China. The voucher specimen (NO. NBGJIB-Ilex-0016) was deposited in the Institute of Botany, Jiangsu Province, and Chinese Academy of Science. Total genomic DNA was extracted using the GMS16011.2.1 Kit (Genmed Scientifics Inc., Wilmington, DE). Genome sequencing was performed on the Illumina NovaSeq platform (Illumina, San Diego, CA) with paired-end reads of 350 bp. In total, 3838 Mb of raw data (3632.4 Mb clean data) were obtained. De novo genome assembly and annotation were conducted by NOVOPlasty (Dierckxsens et al. 2017) and GeSeq (Tillich et al. 2017), respectively. The annotated chloroplast genome was deposited in GeneBank of NCBI (accession number: MT471318).

A typical quadripartite structure was found in the chloroplast genome of *I. chinensis* with a length of 157,885 bp. Two inverted repeats (IRs, including IRa and IRb) of 26,104 bp each, separated by a large single-copy (LSC) region of 87,289 bp and a small single-copy (SSC) region of 18,388 bp, respectively. The *I. chinensis* chloroplast genome contained 135 unique genes, including 37 transfer RNA genes, eight ribosomal *RNA* genes, and 90 protein-coding genes. In the IR regions, 19 genes were found duplicated, including eight protein-coding genes, seven *tRNA* genes and four *rRNA* genes. A total of 13 genes contained one (11 genes) or two (*ycf3* and *clpP*) introns. The overall GC content of the cp genome was 37.61%.

To determine the phylogenetic status of *I. chinensis*, 14 other chloroplast genome sequences were obtained from the Genebank database (Cascales et al. 2017; Park et al. 2019; Yao et al. 2016). A phylogenetic tree was constructed based on the complete cp genome of *I. chinensis* and other reference genomes. The maximum likelihood (bootstrap repeat is 1000) was used for constructing phylogenetic trees using PhyML version 3.0 (http://www.atgc-montpellier.fr/phyml/) (Liu et al. 2019). The phylogenetic tree showed that *I. chinensis* clustered into the Paltoria section, and has more closely related to *I. szechwanensis* and *I. viridis* species (Figure 1). The chloroplast genome sequence of *I. chinensis* in this study will be useful for further analysis on molecular markers and molecular breeding.

**Figure 1.**
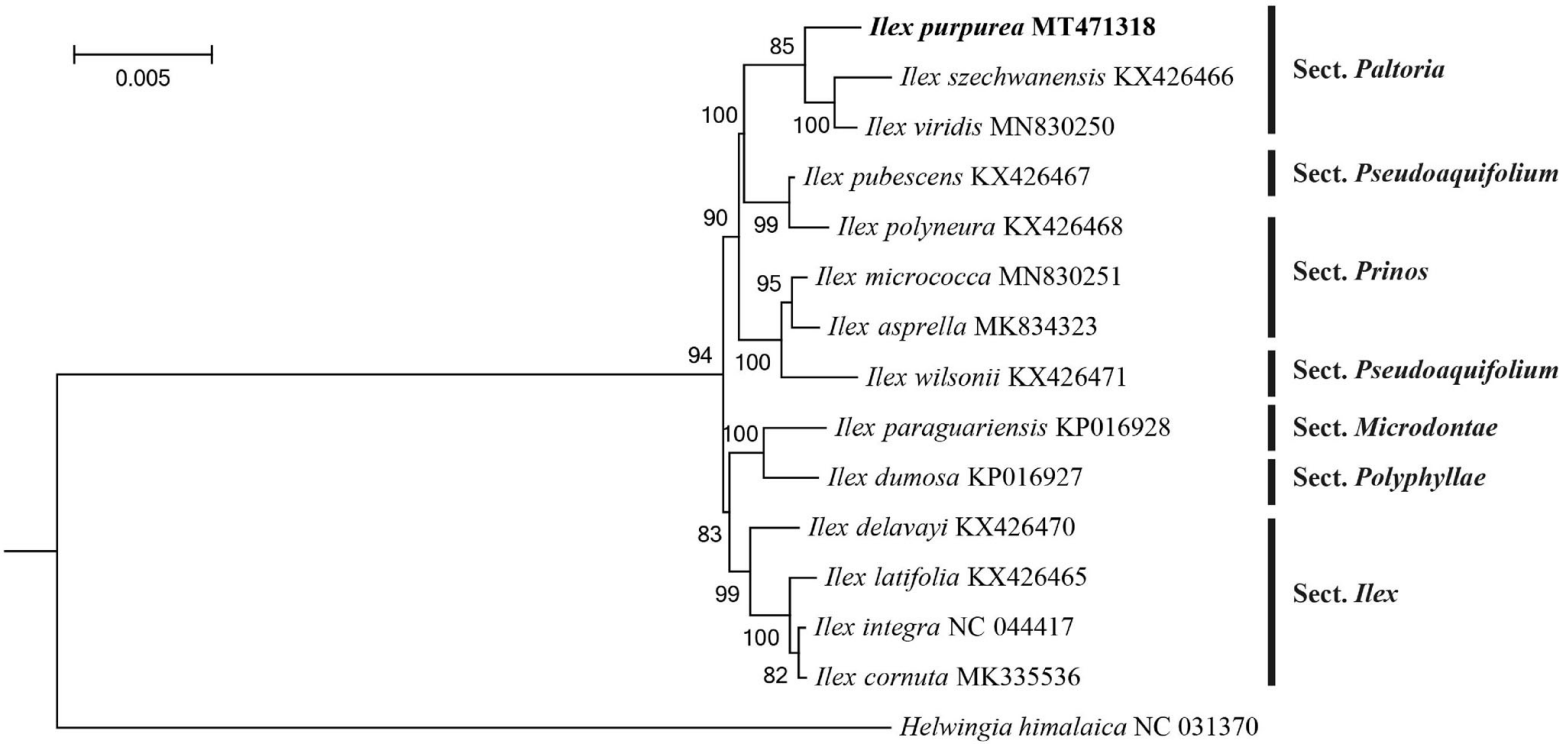
Maximum-likelihood phylogenetic tree based on the sequences of *I. chinensis* and other 14 complete chloroplast genomes. Section names were displayed in the right side of phylogenetic tree (Su et al. 2020). Numbers on the nodes indicate bootstrap values.

## Data Availability

The complete chloroplast genome sequence of *Ilex chinensis* is deposited in the GenBank of NCBI (https://www.ncbi.nlm.nih.gov/) database under the accession number MT471318. The associated Bioproject number is SRR13245417.
